# Cognitive effects of dual‐task training in institutionalized older adults

**DOI:** 10.1002/alz70860_102123

**Published:** 2025-12-23

**Authors:** Clara Burgo, Maria Campos‐Magdaleno, Carlos Dosil‐Díaz, Alba Felpete, Sonali Arora, David Facal

**Affiliations:** ^1^ Departamento de Psicoloxía Evolutiva e da Educación, Universidade de Santiago de Compostela, Santiago de Compostela, Galicia, Spain; ^2^ Instituto de Psicoloxía (IPsiUS), Universidade de Santiago de Compostela, Santiago de Compostela, Galicia, Spain

## Abstract

**Background:**

Older adults' difficulty in performing multiple tasks simultaneously is well documented and has been proposed as an early marker of Alzheimer's disease and dementia. Dual‐task (DT) training has been shown to produce cognitive, emotional and functional improvements in young and older adults and has been proposed as a relevant intervention for long‐term care centers (LTCC) where frailty and cognitive impairment are high, and residents' activity levels are low.

**Method:**

94 older adults (mean = 86.53±8.00 years) from 10 LTCCs in the province of Lugo (Galicia, NW Spain) participated in the study. 46 were assigned to the DT training, while 48 were assigned to the control group and received the usual cognitive stimulation. Information collected before and after the intervention included cognitive (MoCA; a paper‐and‐pencil task in which participants had to follow a path through circles, and an ideational fluency task, both applied in single and dual conditions), affective (Rosemberg Self‐Esteem Scale and Geriatric Depression Scale) and functional (Timed‐Up and Go and grip strength measured with dynamometer) tests.

The DT training consisted of 10 structured sessions delivered weekly by trained staff of the LTCCs. The DTs included physical‐cognitive, physical‐physical and cognitive‐cognitive tasks and eventually some triple tasks. The sessions lasted 50 minutes and were structured in four parts: warn‐up, explanation, training and calm down. The combination of DT tasks was always in the same order: (1) two‐three physical‐cognitive tasks; (2) two‐three physical‐physical tasks; and (3) two‐three cognitive‐cognitive tasks.

GLM repeated measures were run for each cognitive variable as the dependent variable, with a between‐factor Group (DT training‐control), a within‐factor Time (baseline‐post‐test), and affective and functional measures as covariates.

**Result:**

A significant Group x Time interaction was found in the dual conditions of the paper‐and‐pencil (F [1,88]=11.15; *p* <0.01; partial η^2^=0.11) and the fluency tasks (F [1,88]=22.05; *p* <0.001; partial η^2^=0.20), and in the MoCA test score (F [1,88]=13.26; *p* <0.001; partial η^2^=0.13). None of the covariates were significant.

**Conclusion:**

Trained older adults showed significant gains compared to controls, and the improvement generalized to cognitive status, regardless of participants' affective and functional status.

